# Predictions of tDCS treatment response in PTSD patients using EEG based classification

**DOI:** 10.3389/fpsyt.2022.876036

**Published:** 2022-06-29

**Authors:** Sangha Kim, Chaeyeon Yang, Suh-Yeon Dong, Seung-Hwan Lee

**Affiliations:** ^1^Department of Information Technology Engineering, Sookmyung Women's University, Seoul, South Korea; ^2^Clinical Emotion and Cognition Research Laboratory, Inje University, Goyang, South Korea; ^3^Department of Psychiatry, Ilsan-Paik Hospital, Inje University, Goyang, South Korea; ^4^Bwave Inc., Goyang, South Korea

**Keywords:** tDCS, PTSD, EEG, therapeutics, stimulation, machine learning

## Abstract

Transcranial direct current stimulation (tDCS) is an emerging therapeutic tool for treating posttraumatic stress disorder (PTSD). Prior studies have shown that tDCS responses are highly individualized, thus necessitating the individualized optimization of treatment configurations. To date, an effective tool for predicting tDCS treatment outcomes in patients with PTSD has not yet been proposed. Therefore, we aimed to build and validate a tool for predicting tDCS treatment outcomes in patients with PTSD. Forty-eight patients with PTSD received 20 min of 2 mA tDCS stimulation in position of the anode over the F3 and cathode over the F4 region. Non-responders were defined as those with less than 50% improvement after reviewing clinical symptoms based on the Clinician-Administered DSM-5 PTSD Scale (before and after stimulation). Resting-state electroencephalograms were recorded for 3 min before and after stimulation. We extracted power spectral densities (PSDs) for five frequency bands. A support vector machine (SVM) model was used to predict responders and non-responders using PSDs obtained before stimulation. We investigated statistical differences in PSDs before and after stimulation and found statistically significant differences in the F8 channel in the theta band (*p* = 0.01). The SVM model had an area under the ROC curve (AUC) of 0.93 for predicting responders and non-responders using PSDs. To our knowledge, this study provides the first empirical evidence that PSDs can be useful biomarkers for predicting the tDCS treatment response, and that a machine learning model can provide robust prediction performance. Machine learning models based on PSDs can be useful for informing treatment decisions in tDCS treatment for patients with PTSD.

## 1. Introduction

Traumatic experiences, such as the coronavirus disease 2019 (COVID-19) pandemic, are highly prevalent in modern society (1). Therefore, it is essential to understand how to best help those affected by traumatic events as well as informing effective interventions to reduce their psychosocial impacts (2). Many studies have sought to identify the optimal way to prevent and treat post-traumatic stress disorder (PTSD) (3–5), a series of reactions that can occur after someone has experienced traumatic events.

Transcranial direct current stimulation (tDCS) is a possible alternative treatment modality for addressing PTSD. More specifically, tDCS is a therapeutic tool that normalizes brain function and relieves symptoms by sending weak direct current stimulation to the brain surface through electrodes located on the scalp in order to spontaneously activate nerve cells. This is a safe neuromodulation technique that has few adverse effects (i.e., loss of consciousness, convulsions, abnormal sensations) as compared to other brain stimulation modalities (6).

Previous research has demonstrated that tDCS shows great promise as a therapeutic intervention for treating clinical neuropsychiatric disorders, including PTSD, depression, and cognitive decline. Auditory verbal hallucinations are robustly reduced by tDCS (7). Another study recently suggested that tDCS may be a promising novel treatment for addressing impulsivity in attention deficit hyperactivity disorder (ADHD) (8). A small number of studies have reported clinically significant improvements following tDCS treatment with respect to a range of cognitive and emotional performance metrics in PTSD patients evaluated using electroencephalograms (EEG), event-related potentials (ERP), and alpha peak frequencies (APF) (9).

In the current study, although tDCS treatment resulted in clinical improvements in patients with PTSD, not all patients were positively impacted by tDCS. Several patients showed a clinical response, while others showed no difference or even a worsening of their symptoms. tDCS modulates spontaneous neuronal activity, and the amount and direction of its effects critically depend on the physiological state of the target neural structures. Since the effects of tDCS depend on the baseline status of the brain at the time of application, individual patients show considerable heterogeneity in treatment outcomes (10). Consequently, tDCS responses are highly individualized, and this critically affects the evaluation of tDCS responses (11).

Supervised machine learning methods (e.g., support vector machines; SVM) can be used to identify and predict individual clinical responses in electric field characteristics following tDCS treatment (12). Individual prognostic classifications of tDCS outcomes can provide important insights for future tDCS interventions. However, to the best of our knowledge, there are currently no studies utilizing machine learning strategies in patients with PTSD undergoing tDCS treatment.

EEG has been used as a biomarker for detecting and classifying brain dysfunction. Previous work has demonstrated that various forms of brain disorders, including PTSD (13), schizophrenia (14), major depressive disorder (15) and Alzheimer's disease, can be diagnosed by monitoring patients' EEG responses. Therefore, analyses using EEG responses have the potential to identify clinical responses to neuropsychiatric treatments, including tDCS. This study aimed to demonstrate the potential of evaluating EEG responses in order to classify responders and non-responders among patients with PTSD. The results of this study might inform appropriate individualized treatments that can be adequately introduced by selecting patients based on their characteristics and expected treatment effects.

In this study, we compared electrophysiological responses before and after tDCS treatment by analyzing 62-channel EEG readings in 48 patients with PTSD. We aimed to provide a tool for increasing the effectiveness of tDCS by building and validating a personalized therapeutic response classification model. A machine learning model was trained to determine the best performing EEG channels and frequency bands. These features were also used to predict the outcomes of tDCS treatment in patients with PTSD. We hypothesized that (1) a statistically significant difference in the specific channel and frequency band that can be observed by comparing EEG responses before and after treatment between responders and non-responders, and (2) therapeutic responders and non-responders could be predicted and classified using the EEG features identified in (1).

## 2. Materials and methods

### 2.1. Participants

Fifty-one patients with PTSD were enrolled in this study. Patients were diagnosed by an experienced psychiatrist using the Structured Clinical Interview for DSM-V (SCID) Axis I Psychiatric Disorders (16). The Clinician-Administered PTSD Scale for DSM-5 (CAPS-5) was used to evaluate psychiatric symptoms (17, 18). Participants aged <19 years or those with too many EEG artifacts due to body and eye movements were excluded from the current study. A total of 48 patients (23 males, age 50.81 ± 11.60 years [mean ± SD]) were ultimately enrolled. All participants signed a written informed consent form that was approved by the institutional review board of Inje University, Ilsan Paik Hospital (IRB no. 2015-07-025). This study was conducted in accordance with the principles of the Declaration of Helsinki and its later amendments.

We categorized the patients into responders and non-responders based on their CAPS-5 scores. For the clinical assessment, total severity scores (Sev) and the total number of prevalent PTSD symptoms (Sx) were obtained for each patient. These scores statistically significantly decreased in both groups after tDCS stimulation [Sev: *t*_(47)_ = 6.33, *p* < 0.001, Sx: *t*_(47)_ = 6.89, *p* < 0.001]. Patients whose PTSD scores (total Sev scores and total Sx scores) improved by more than 50% were designated responders. The remaining participants were designated non-responders. In total, 31 participants were designated non-responders and the remaining 17 participants were designated responders.

Demographic data for the participants in each group are presented in Table 1. We additionally conducted comparisons between responders and non-responders on the different sub-scales of the main PTSD symptoms. The sub factors of PTSD symptoms in CAPS-5, intrusions (B), avoidance (C), negative affect and anhedonia (D) and externalizing, anxious arousal and dysphoric arousal (E), were obtained for comparison. Independent *t*-tests were employed to compare age, educational attainment, and Sev and Sx scores and other main scales across groups. Sev and Sx scores and other scales include pre- and post-treatment values measured before and after treatment, respectively.

**Table 1:** Demographic and clinical characteristics of responders and non-responders.

	**Responders (*****N*** **= 17)**	**Non-responders (*****N*** **= 31)**	***p***
Age (years)	51.18 ± 10.84	50.61 ± 12.17.	0.874
Sex			0.489
Male	7 (41.2)	16 (51.6)	
Female	10 (58.8)	15 (48.4)	
Education	10.59 ± 4.37	11.65 ± 3.27	0.348
(years)			
CAPS-5			
Pre			
B Sev	11.76 ± 5.95	10.77 ± 4.92	0.539
B Sx	3.47 ± 1.70	3.58 ± 1.52	0.973
C Sev	5.71 ± 2.49	3.48 ± 2.42	0.004
C Sx	1.59 ± 0.62	1.19 ± 0.83	0.115
D Sev	13.65 ± 6.59	13.16 ± 6.16	0.8
D Sx	4.06 ± 1.52	4.10 ± 1.80	0.815
E Sev	11.65 ± 4.43	10.35 ± 4.56	0.348
E Sx	3.53 ± 1.07	3.52 ± 1.39	0.973
Total Sev	42.76 ± 16.22	37.42 ± 13.29	0.224
Total Sx	12.65 ± 3.69	12.26 ± 4.07	0.745
Post			
B Sev	2.29 ± 2.17	7.87 ± 3.86	<0.001
B Sx	0.65 ± 0.79	2.61 ± 1.54	<0.001
C Sev	0.53 ± 1.23	4.32 ± 2.69	<0.001
C Sx	0.24 ± 0.56	1.55 ± 1.12	<0.001
D Sev	2.88 ± 2.87	10.77 ± 5.70	<0.001
D Sx	0.71 ± 0.92	3.42 ± 1.84	<0.001
E Sev	4.24 ± 2.82	7.13 ± 3.42	0.005
E Sx	1.35 ± 1.00	2.48 ± 1.43	0.008
Total Sev	9.94 ± 6.02	30.1 ± 9.64	<0.001
Total Sx	2.94 ± 2.3	10.06 ± 3.56	<0.001

*PTSD, post-traumatic stress disorder; CAPS-5, clinician-administered PTSD scale for DSM-5; Sev, severity; Sx, symptoms; B, intrusions factor of CAPS-5; C, avoidance factor of CAPS-5; D, negative affect and anhedonia factor of CAPS-5; E, externalizing, anxious arousal and dysphoric arousal factor of CAPS-5*.

### 2.2. tDCS protocol and application

Each tDCS session was applied using two saline-soaked sponge pads, with the anodal electrode positioned over the dorsolateral prefrontal cortex (with a F3 electrode location selected according to the International 10/20 System) and a cathode electrode placed over the F4 electrode. The position of the anode (F3) electrode montage is important in patients with posttraumatic stress disorder (PTSD) as it is closely related to the left dorsolateral prefrontal cortex (DLPFC). DLPFC plays a central role in emotional processing by regulating fear expression through projections to the vmPFC (19) and lateralized DLPFC dysfunction could be the underlying cause of stress and memory problems shown in PTSD patients (20). In addition, patients with PTSD showed weakly connected and hypoactive central executive network (CEN) where DLPFC is involved as a major node (21). We further applied tDCS on athode (F4) as the position determines current intensity of stimulation at the left DLPFC by affecting neuromodulation under the anode (22). Therefore, considering (a) previous studies supporting the relationship between abnormalities found in PTSD patients and DLPFC dysfunction (23, 24) and (b) the efficacy of tDCS for PTSD on DLPFC (25), this study involved right and left DLPFC (F3 and F4) as the stimulation area of tDCS for patients with PTSD.

The active stimulation protocol involved applying 2.0 mA intensity for 20 min. Participants sat quietly during the stimulation session, while the researcher monitored and recorded tDCS electrode impedances. Each subject received 1 tDCS session per day during 10 days. Thus, total 10 session of tDCS were applied for a subject.

### 2.3. EEG data acquisition

For EEG acquisition, the participants were seated in a slightly dim room for 3 min with their eyes-closed and in a relaxed state. During the experiment, all the participants were told in advance not to move or sleep. EEG data were acquired using a NeuroScan SynAmps amplifier (Compumedics USA, Charlotte, NC, USA), and a NeuroScan Quick-Cap with 62 Ag/AgCl electrodes was placed according to the extended international 10–20 system. All recorded EEG data were sampled at 1,000 Hz and filtered using a 0.1–100 Hz bandpass filter. The electrode impedance was kept below 5 kΩ, with ground and reference electrodes located on the forehead and Cz reference point, respectively.

### 2.4. EEG preprocessing

Raw EEG data from 62 EEG channels (excluding HEO, VEO, and EKG channels) were preprocessed using EEGLAB (26) and MATLAB R2020a software (MathWorks, Natick, MA, USA). EEG data sampled at 1,000 Hz were re-referenced according to the common average reference and the baseline data was removed. A Butterworth bandpass filter was used to filter the EEG data with cutoff frequencies of 1 and 50 Hz. Artifacts caused by movements, such as those of the muscles and eyes, were rejected using a independent component analysis (ICA). More specifically, all independent components were filtered by visual inspection and segments containing large artifacts were excluded. After pre-processing, EEG segments with a length of 150 s were prepared based on data from 48 patients.

For each segment, power spectral density (PSD) was extracted using Welch's method (27). Welch PSD values were obtained using the built-in method in MATLAB (28, 29). More specifically, data were divided into several 1 s segments with 50% overlap. Next, each segment was windowed using the Hamming window and the periodogram of each windowed semgent was obtained after a fast Fourier transform. Finally, all periodograms were averaged to obtain the Welch PSD values for each participant using the average powers in five specific frequency bands: delta (1–4 Hz), theta (4–8 Hz), low alpha (8–10 Hz), high alpha (10–12 Hz), and beta (12–30 Hz).

### 2.5. Feature extraction

**Figure 1:**
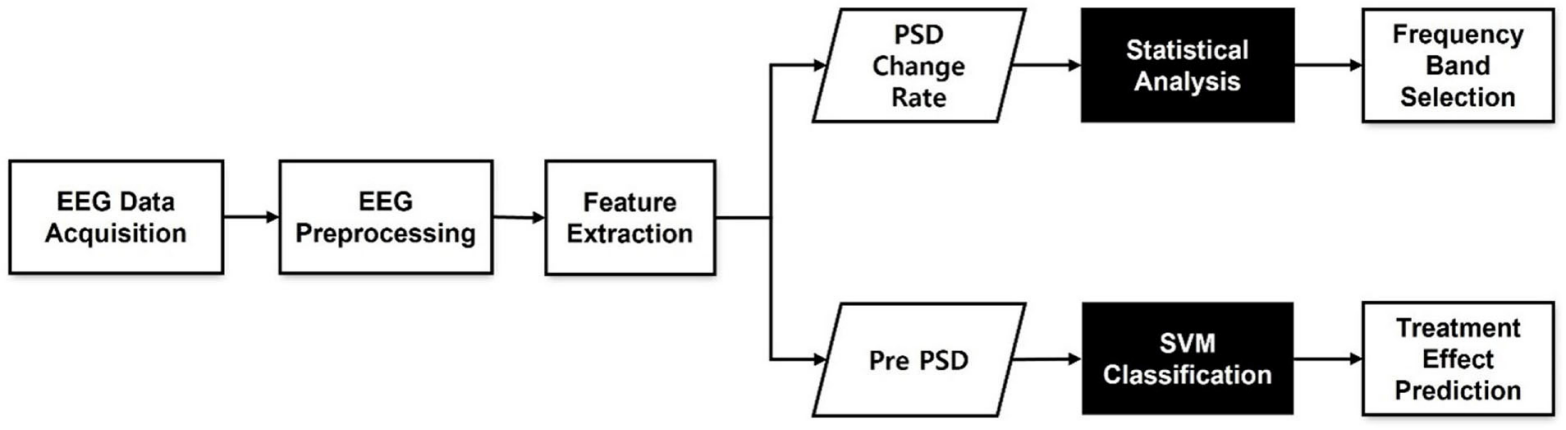
The flowchart of overall analysis procedure.

Figure 1 summarizes the analytical procedure used in this study. First, we investigated treatment outcomes by comparing PSD values before and after tDCS treatment, as shown in the upper pathway in Figure 1. To assess differences, we computed PSD change rates after treatment. Changes in the frequency band power over the treatment period were calculated using the following equation:


(1) $$\begin{array}{r} P_{change}\left(f\right)=\frac{P_{pre}\left(f\right)-P_{post}\left(f\right)}{P_{pre}\left(f\right)} \end{array}$$

where *P*_*pre*_(*f*) is the pre-treatment PSD in a certain frequency band, and *P*_*post*_(*f*) is the post-treatment PSD in the same frequency band. By comparing *P*_*change*_(*f*) between responders and non-responders, we attempted to identify frequency bands that exhibited therapeutic effects and used these bands to predict treatment responses.

To predict treatment response, only pre-treatment PSD was used to classify responders and non-responders. Pre-PSD was used as the input to the classifier, as shown in the lower path in Figure 1.

### 2.6. Statistical analysis

An independent *t*-test was used to evaluate the mean difference in PSD change rates between responders and non-responders. Since the number of responders was less than 30, we performed the Kolmogorov-Smirnov test for normality and Levene's test for equality of variance. If normality was not satisfied, we employed a Mann-Whitney *U*-test instead of a two-sample *t*-test as appropriate. Moreover, we used multiple EEG channels to compare PSD change rates between the two groups. *P*-values were adjusted using the false discovery rate (FDR) to control for Type I errors. All statistical analyses were performed using R Statistical Software (version 4.1.1; R Foundation for Statistical Computing, Vienna, Austria).

### 2.7. Classification

An SVM was used to classify responders and non-responders using the PSD of the pre-treatment phase. SVM is well-known as a classic supervised learning classifier (30). In this study, the radial basis function was selected as the kernel function in the SVM to model complex nonlinear relationships. We used the grid search method with a range of C and γ values (ranging from 0.001 to 100) to adjust the optimal combination of SVM parameters. One combination of hyperparameters with the best cross-validation accuracy was selected and used to train an SVM on the entire dataset (31). Using each EEG channel's average powers from five frequency bands, we first split the data into training and testing sets for five-fold cross validation. For each fold, a grid search was performed to identify the optimal parameter values producing the best predictive model. Model evaluation was based on evaluations of the area under the receiver operating characteristic (ROC) curve (AUC). We calculated classification performance using the AUC as well as sensitivity and specificity. To avoid imbalance between two classes and get reasonable conclusion, we also computed balanced accuracy which is based on two common metrics, sensitiviy and specificity.

More specifically, we evaluated classification performance based on readings from a single EEG channel for each frequency band. We then improved the SVM model using multiple channels, starting from a single channel and iteratively adding channels one by one. We added a channel that best improved the model until all 62 channels were used for training. We aimed to design EEG channels for each frequency band to provide effective predictors of treatment outcomes. We also performed the cluster-based permutation test to deal with the multiple comparison problem in multi-channel EEG data. We produced 1,000 random permutations and *p*-values were obtained from the best SVM model which showed the highest AUC in multi-channel classification (32).

## 3. Results

### 3.1. Statistical analysis for treatment outcomes

**Figure 2:**
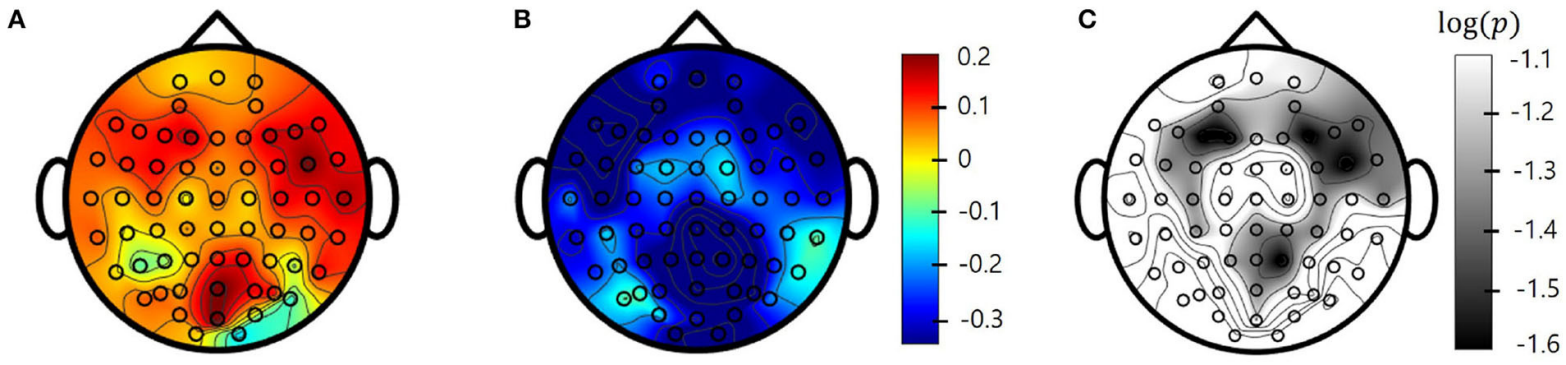
Topographies of beta band averaged power spectral density (PSD) change (pre-post) rates in **(A)** responders and **(B)** non-responders. **(C)** log-scaled adjusted *p*-values (corrected) from false discovery rate (FDR) obtained from two-sample *t*-tests also in Beta band. Values between electrodes are interpolated.

To determine the frequency bands that could best select treatment outcomes following tDCS in patients with PTSD, we compared the rates of change in PSD values (*P*_*change*_(*f*)) between responders and non-responders within five frequency bands. After FDR correction, some channels in the theta and beta bands showed statistically significant differences between groups. None of the channels in the remaining three frequency bands were found to be statistically significant. The theta band had six significant channels (F7, F8, FC6, FT8, P2, and POZ) and the beta band had 23 significant channels (AF3, AF4, F5, F3, F1, F2, F4, F6, F8, FC5, FC4, FC6, FT8, C3, C4, T8, CP3, CPZ, CP2, CP4, PZ, P2, and POZ) at a statistical significance level of 0.05. Although there were statistically significant differences of the rates of change in PSD values (*P*_*change*_(*f*)) in those frequency bands, there were no existing significant differences in baseline power (pre-PSD). The F8 channel in the theta band [*t*_(39.348)_ = –4.18, *p* < 0.001] and the FC6 channel in the beta band [*t*_(42.95)_ = –3.88, *p* < 0.001] showed the strongest statistically significant differences. Figures 2A,B show the topographies of the averaged beta PSD change rates in responders and non-responders in beta frequency. As can be clearly seen in the figure, responders exhibited decreased beta PSD values within all 62 channels after treatment, with greater reductions especially in the frontal and centro-parietal regions. In contrast, in most non-responders, beta PSD values increased after treatment. Figure 2C illustrates the statistical significance of the topography of the logarithmic FDR-corrected *p*-values. Statistical significance was observed in the channels located in the frontal and centro-parietal regions. The FC6 channel in the frontal region, which showed the statistically strongest significant difference, showed a relatively large decrease of PSDs in responders (pre-PSD: 0.481 ± 0.32, post-PSD: 0.331 ± 0.172). On the other hand, it shows relatively large increase in non-responders (pre-PSD: 0.383 ± 0.286, post-PSD: 0.422 ± 0.355).

### 3.2. Prediction of treatment response

**Figure 3:**
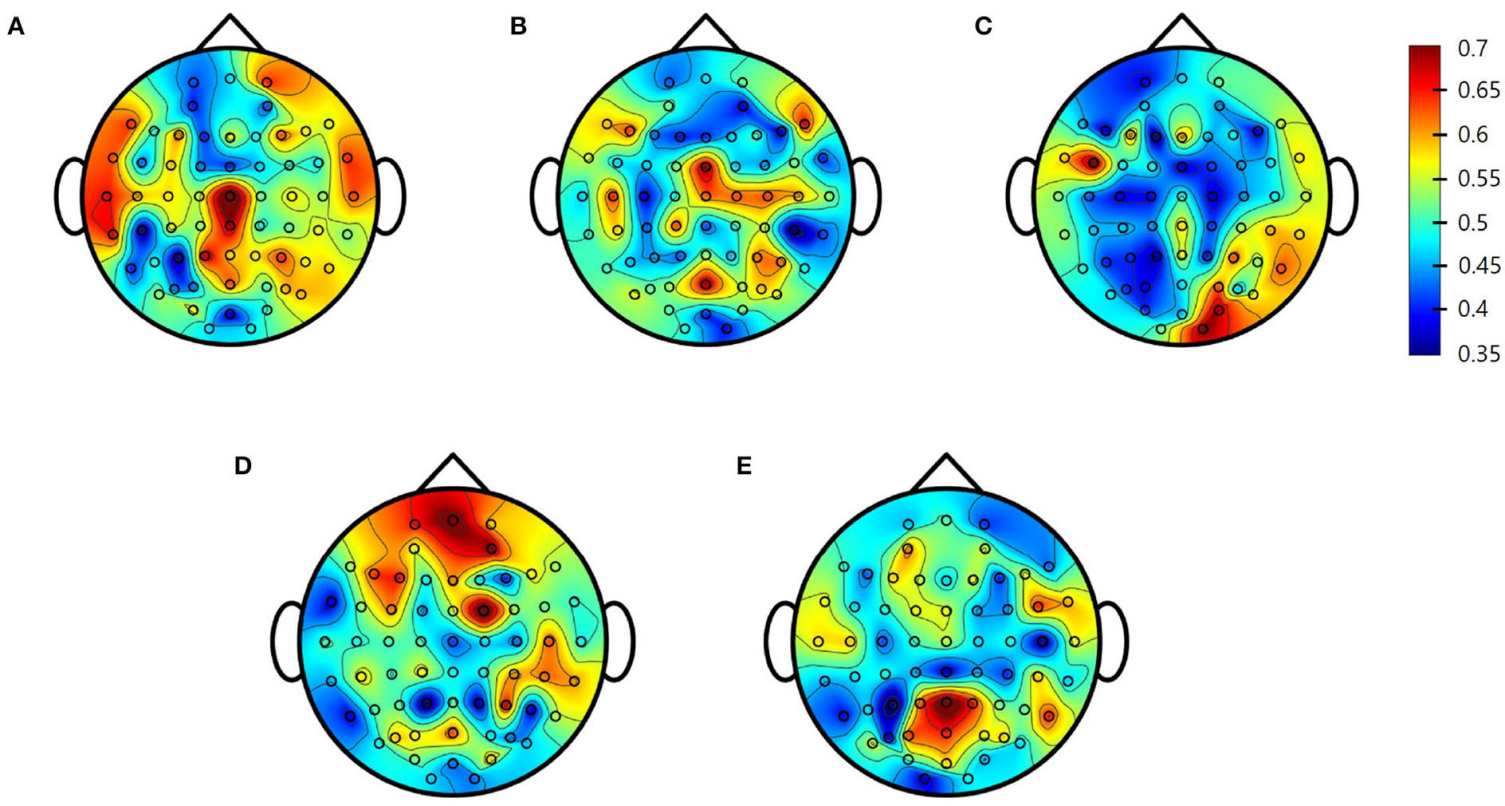
Single-channel support vector machine (SVM) performance (area under the receiving operating characteristics curve [AUC]) for each electrode. **(A)** Delta; **(B)** Theta; **(C)** Alpha Low; **(D)** Alpha High; **(E)** Beta.

To determine whether pre-PSD reading could be used to predict treatment response, an SVM was trained to classify responders and non-responders. First, as a single-channel approach, the SVM model was trained for each channel and frequency band to identify the channels and bands that best represented the treatment response. SVM classification of all five bands accurately distinguished tDCS responders from non-responders, with AUCs ranging from 0.71 to 0.81 (delta: AUC = 0.81 at Cz; theta: AUC = 0.71 at FCz; alpha low: AUC = 0.72 at FC5; alpha high: AUC = 0.79 at FC2; beta: AUC = 0.78 at Pz). Figure 3 shows the topographies of the SVM classification performances in terms of the AUC for each frequency band.

**Figure 4:**
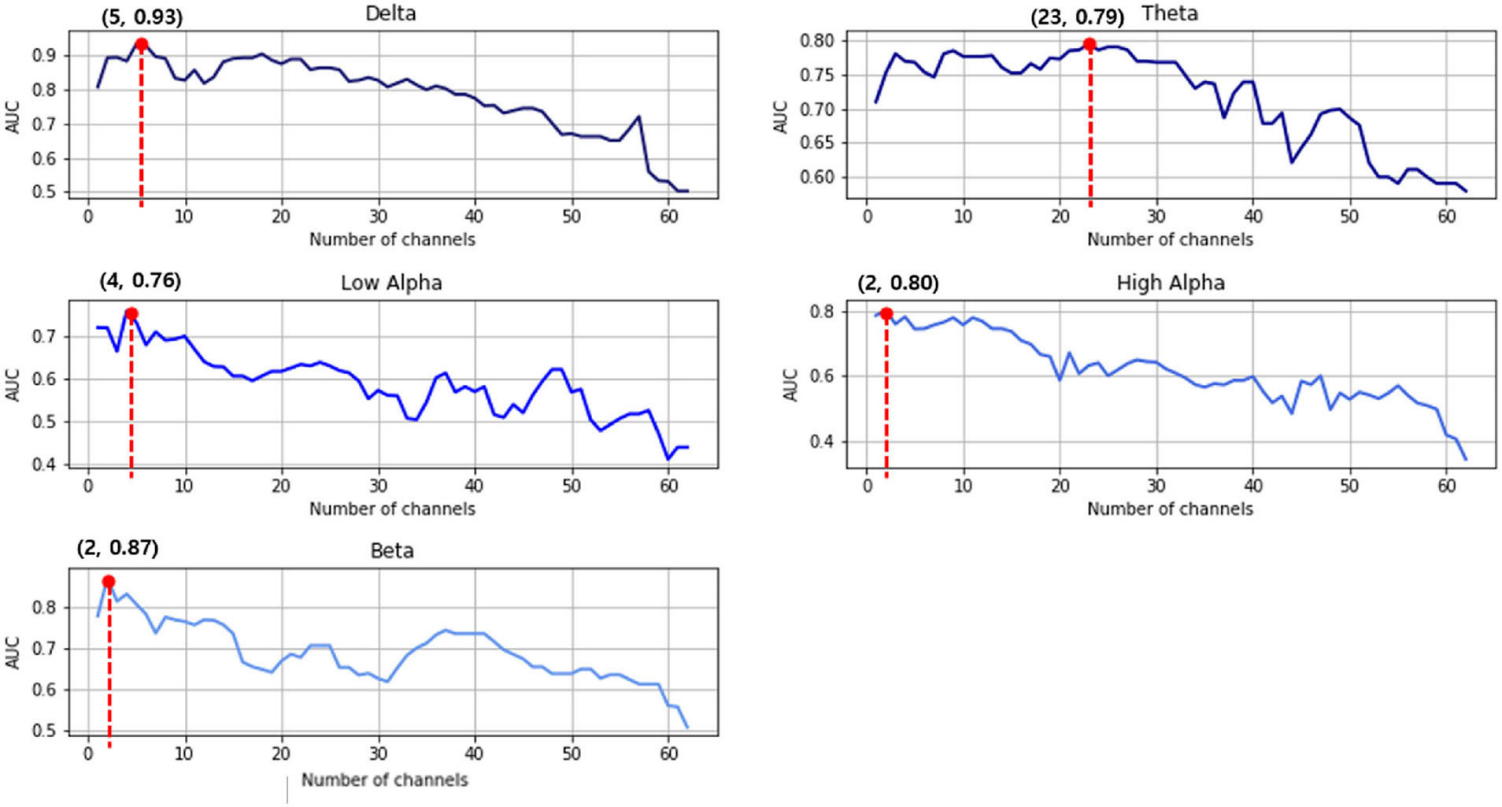
Support vector machine (SVM) prediction accuracy in the area under the receiving operating characteristics curve (AUC) for each frequency band. The red dots indicate the highest classification results of that frequency range.

Second, the multichannel approach improved the SVM classification between the two groups to a greater degree. More specifically, as shown in Figure 4, all five frequency bands usually demonstrate that the AUC increases for the first few channels and then continues to decrease as the number of channels increase. Therefore, the prediction accuracy reached its maximum in all frequency bands when using this multichannel approach. For the delta example, the single best prediction accuracy was 0.81 at Cz. However, adding the O1, FC2, FC1, and F2 channels provided a much better performance at 0.93. Table 2 summarizes the prediction performance of the single-and multi-channel approaches in each frequency band.

**Table 2:** Support vector machine (SVM) classification results for the best channel per frequency range with pre-power spectral density (PSD) readings.

**Frequency** **band**	**Single max**					**Multiple max**					
	**Channel**	**AUC**	**Sensitivity**	**Specificity**	**Balanced** **accuracy** **(%)**	**Channel**	**AUC**	**Sensitivity**	**Specificity**	**Balanced** **accuracy** **(%)**	***p*** **-value**
Delta	CZ	0.81	0.7	0.8	75.2	CZ, O1, FC2, FC1, F2	0.93	0.77	0.87	81.7	<0.001
Theta	FCZ	0.71	0.35	0.81	58.0	FCZ, POZ, CPZ, FZ, FC4, FC3, F6, FC1, FC2, CP1, T8, AF4, CP2, CP4, F4, F2, C2, CZ, P6, CP5, FC5, P7, C6	0.79	0.12	0.97	54.2	0.006
Alpha Low	FC5	0.72	0.32	0.91	61.5	FC5, CP6, P2, PZ	0.76	0.13	0.93	53.3	0.011
Alpha High	FC2	0.79	0.53	0.87	70.2	FC2, P4	0.8	0.32	0.83	57.5	0.04
Beta	PZ	0.78	0.37	0.81	59.0	PZ, CP5	0.87	0.73	0.78	80.0	0.002

*AUC, area under the receiver operating characteristics curve*.

## 4. Discussion

The present study investigated tDCS treatment responses using statistical analysis and machine learning techniques for EEG data in patients with PTSD. Each individual was defined as a responder or non-responder to tDCS treatment depending on psychiatric symptom changes assessed by the CAPS-5 evaluation. Since we acquired multi-channel EEG data before and after tDCS treatment, a comparison could easily be made to find the EEG feature best predicting responsiveness. Using the pre-delta PSDs for five selected channels (Cz, O1, FC2, FC1, and F2), the SVM model presented in this paper was able to predict an individual's tDCS responsiveness with an AUC of 0.93, despite the small size of the training data.

### 4.1. EEG for monitoring tDCS

Together with electrical stimulation, EEG monitoring can provide additional mechanistic information as well as information regarding the clinical effects on brain function. EEG has been widely used to measure the cortical effects of tDCS. For example, Boonstra et al. (33) presented changes in mean frequency, demonstrating that the mean frequency was statistically significantly reduced after tDCS stimulation as compared to sham stimulation. Song et al. (34) observed a statistically significant increase in beta power during stimulation. Cavinato et al. (35) observed changes in cortical EEG oscillations, such as alpha and beta waves, in patients with disorders of consciousness. Similarly, we observed theta and beta power changes that were similar to other studies demonstrating spectral differences in PTSD patients with tDCS treatment (36, 37). These two frequency bands, theta and beta, have been reported as the key observable indices for clinical effects of tDCS treatment related to major symptoms of PTSD, such as stress, depressive and anxiety. According to Palacios-García's study, the increase in psychosocial stress and stress-related anxiety was related to specific changes in beta-band (38). Dunkley also reported theta band plays a critical role in attentional, depressive and anxiety-related sequelae observed in PTSD populations (39). Therefore, our findings clinically suggest that the alleviation of PTSD symptoms, which is the effect of tDCS treatment, can be observed and monitored in patients' EEG.

Most of the aforementioned studies reported an increase in spectral power after stimulation, and this result was replicated in our study. However, when the participants were divided into responders and non-responders, this trend was observed only for non-responders. More specifically, the non-responders showed an increase, whereas the responders showed a decrease after treatment. As there were statistically significant group differences in PTSD Total sev and sx scores after treatment, as well as some sub scales of the CAPS (B, C, D and E), these spectral power differences could be related to clinical effects to PTSD in major symptoms such as intrusions, avoidance, negative affect and anhedonia. Especially in a key disease factor (avoidance, *P* = 0.004), the responder group differed significantly from the non-responder group, with a large effect size. Both groups present differently clinicaly at baseline. This avoidance factor in CAPS-5 is mainly related to the functional deterioration of the left frontal lobe (40, 41). The left frontal anodal tDCS performed in our study is a left frontal lobe activating protocol. Therefore, these numerical differences in the treatment responders suggest that the effect of tDCS treatment was clearly applied in the treatment responders.Since evaluating differences between responders and non-responders may help in identifying patients responsive to tDCS at early stages of treatment, it is crucial to compare patient groups through EEG monitoring.

### 4.2. Machine learning for predicting responsiveness

Owing to recent advances in machine learning, clinical outcomes can now be measured or evaluated using electrophysiological data. For instance, a study by Zandvakili et al. (42). presented an approach for predicting the clinical response to brain stimulation in mental disorders using resting EEG readings. Specifically, these researchers proposed an automated EEG classification to predict tDCS treatment outcomes in patients with major depressive disorder (MDD). Based on their proposed cognition labels, the evaluated machine learning classifier exhibited a high predictive performance (87%) using a single channel and an even higher predictive performance (92%) using multiple channels. Albizu et al. also reported an SVM model that could predict individual tDCS responsiveness with 86% accuracy (12).

Similar to the study conducted by Zandvakili et al, we evaluated prediction performance while comparing single channels and multiple channels. By comparing the performance of each of the five frequency bands, it was possible to identify specific channels and bands with high prediction performance. The proposed approach demonstrated that it is possible to predict therapeutic outcomes using resting EEG readings with relatively high performance and accuracy (AUC = 0.93). This result showed higher predictive performance than the case of logistic regression was performed with the baseline avoidance scale (AUC = 0.75), a key disease factor that showed a significant difference between the two groups in baseline. Furthermore, in each frequency band, EEG electrodes located in the middle line (e.g., FCZ, CZ, PZ) and electrodes placed in frontal region (e.g., FC5, FCZ, FC2) commonly showed the highest performance. The accurate prediction of tDCS response is meaningful because the efficiency of clinical treatment can be substantially increased given this information.

### 4.3. Study limitations

Despite the high performance of the predictions generated in this study, we acknowledge some limitations of this work. For example, the sample data were based on subjects who were diverse in age, with patients ranging in age from their 20s to their 70s. According to Bokszczanin's study, treatment outcomes may vary according to age and gender (43). However, differences in age-specific effects of tDCS treatment were not clearly observed in this study. In addition, the CAPS-5-based patient group assignments may not be divided clearly. As the degree of improvement varies substantially from person to person, some people are located near the boundary between response and nonresponse. We anticipate extending our study to a much larger and more comprehensive study population of patients with PTSD so that the therapeutic effects of tDCS can be comprehensively identified and predicted.

## 5. Conclusions

The current study investigated tDCS treatment responsiveness in patients with PTSD using EEG spectral power and machine learning-based prediction methods. In this study, the evaluated patients in the two groups showed statistically significant differences in EEG spectral power in the theta and beta frequency bands with respect to their treatment response. In addition, we demonstrated that machine-learning-based classifications can predict tDCS treatment outcomes with considerable accuracy. From these results, it is possible to identify specific channels and bands that most accurately represent the tDCS response in patients with PTSD. Despite one of the aforementioned limitations (i.e., that we only considered two labels for CAPS-5), we conclude that these results have the potential to hold new insights as a basis for diagnosing and predicting the clinical response to tDCS treatment. These results could therefore provide critical information informing a meaningful approach for the early identification of patients who might be clinically affected by tDCS treatment, thus reducing the cost and time these patients would otherwise expend during the treatment process. Our findings inform future research directions, and, if confirmed, are expected to ultimately inform medical guidelines.

## Data availability statement

The datasets presented in this article are not readily available because of ethical concerns. Requests to access the datasets should be directed to S-HL, lshpss@hanmail.net.

## Ethics statement

The studies involving human participants were reviewed and approved by the Institutional Review Board of Inje University, Ilsan Paik Hospital (IRB no. 2015-07-025). The patients/participants provided their written informed consent to participate in this study.

## Author contributions

SK: data analysis and writing-original draft. CY: data collection and writing-review and editing. S-YD: conceptualization, funding acquisition, and writing-review and editing. S-HL: conceptualization, supervision, and writing-review and editing. All authors contributed to the article and approved the submitted version.

## Funding

This research was supported by KBRI Basic Research Program through Korea Brain Research Institute funded by Ministry of Science and ICT (22-BR-02-02), the Commercialization Promotion Agency for R&D Outcomes (COMPA) funded by the Ministry of Science and ICT (MSIT) (Commercialization of health functional foods by verifying the efficacy of functional ingredients and developing the selection method of appropriate content based on AI), and by Sookmyung Women's University Research Grants (1-2203-2006).

## Conflict of interest

S-HL was employed by Bwave Inc. The remaining authors declare that the research was conducted in the absence of any commercial or financial relationships that could be construed as a potential conflict of interest.

## Publisher's note

All claims expressed in this article are solely those of the authors and do not necessarily represent those of their affiliated organizations, or those of the publisher, the editors and the reviewers. Any product that may be evaluated in this article, or claim that may be made by its manufacturer, is not guaranteed or endorsed by the publisher.

## Abbreviations

ADHD: attention deficit hyperactivity disorder
APF: alpha peak frequency
AUC: area under the ROC curve
CAPS-5: Clinician-Administered PTSD Scale for DSM-5
COVID-19: coronavirus disease 2019
EEG: electroencephalogram
ERP: event-related potentials
FDR: false discovery rate
ICA: independent component analysis
PSD: power spectral density
PTSD: post-traumatic stress disorder
ROC curve: receiver operating characteristic curve
SCID: Structured Clinical Interview
Sev: total severity score
Sx: total number of prevalent PTSD symptoms
SVM: support vector machines
tDCS: transcranial direct current stimulation.
